# Using heart rate variability to predict neurological outcomes in preterm infants: a scoping review

**DOI:** 10.1038/s41390-024-03606-5

**Published:** 2024-10-05

**Authors:** Magdalena Smolkova, Shivani Sekar, Seh Hyun Kim, John Sunwoo, Mohamed El-Dib

**Affiliations:** 1 School of Clinical Medicine, University of Cambridge, Cambridge, UK; 2 Division of Newborn Medicine, Department of Pediatrics, Brigham and Women’s Hospital, Harvard Medical School, Boston, MA USA; 3 Division of Neonatology, Department of Pediatrics, Seoul National University Children’s Hospital, Seoul, Republic of Korea; 4 Athinoula A. Martinos Center for Biomedical Imaging, Massachusetts General Hospital, Harvard Medical School, Boston, MA USA

## Abstract

**Abstract:**

Infants born preterm are at higher risk of neurological complications, including intraventricular haemorrhage and white matter injury. After discharge, these infants may experience adverse neurodevelopmental outcomes and exhibit lower educational attainment. Early detection of brain injury and accurate prediction of neurodevelopmental impairment would allow early intervention and support. Heart rate variability (HRV) describes the variation of time intervals between each subsequent heartbeat. HRV is controlled by the autonomic nervous system, which may be affected by hypoxia and compromised blood flow. While HRV has primarily been investigated in neonatal sepsis, the association between HRV, brain injury and neurodevelopmental outcomes in preterm infants is less established. The present scoping review examines the utility of HRV monitoring for predicting short-term and long-term neurological outcomes in preterm infants. Following systematic search of Medline, Embase, Web of Science and the Cochrane Library, 15 studies were included. Nine studies examined the relationship between HRV and brain injury, with all but two showed an association. Eight studies examined the relationship between HRV and long-term outcomes and all eight found an association. This scoping review suggests that decreased HRV in the neonatal period is associated with short- and long-term neurodevelopmental outcomes in preterm infants.

**Impact:**

- Changes in heart rate variability correlate with the occurrence of intraventricular haemorrhage in preterm infants.
- A decrease in heart rate variability may precede the development of intraventricular haemorrhage.
- Alterations in heart rate variability correlate with long-term neurodevelopmental outcomes.
- Significant variability exists in metrics used in assessing heart rate variability.

## Introduction

Preterm birth is associated with various adverse outcomes, which tend to be less severe with increasing gestational age.^1^ The most acute complications of preterm birth include respiratory distress syndrome, apnoea of prematurity, metabolic immaturity, difficulty feeding and a predisposition to various infections.^2^ Preterm infants are also at risk of neurological complications including germinal matrix haemorrhage, intraventricular haemorrhage (IVH) and white matter injury (WMI).^3^

IVH occurs in about 25% of preterm infants with very low birthweight.^3^ Blood accumulation from IVH may damage adjacent structures. When significant and associated with post-haemorrhagic ventricular dilatation, it can lead to further damage through mass effect and increased intracranial pressure, impairing brain perfusion.^4^ Higher grades IVH are associated with increased rates of cerebral palsy (CP), neurosensory impairment, impaired cognitive ability, blindness and hearing loss.^3^

WMI in preterm infants is subdivided into focal cystic necrosis, focal microscopic necrosis and diffuse non-necrotic lesions. While the first is strongly associated with the development of CP, the latter two are associated with impaired academic performance and lower IQ.^3^

Extremely preterm infants, with and without brain injury, also have increased rates of disabilities including CP, deafness, cognitive impairment and blindness. Moreover, they show higher rates of behavioural difficulties and executive dysfunction or attention deficit hyperactivity disorder.^5–8^ Early detection of brain injury or the potential for adverse neurodevelopment is essential for effective management. The diagnosis and management of IVH may prevent the progression of brain injury and improve outcomes.^9^ Furthermore, early developmental interventions have been shown to improve motor and cognitive development in preterm infants.^10^ Reliable early markers of brain injury and increased risk of neurodevelopmental impairment in the neonatal period are needed to allow better risk classification and potential early interventions.

Heart rate variability (HRV) describes the changes in heart rate (HR) over time, reflecting the activity of the autonomic nervous system, combining the opposing influences of the sympathetic and parasympathetic nervous systems.^11,12^ In adult patients, decreased HRV has been associated with increased all-cause mortality.^13^ HRV has been shown to decrease in pathological states including severe infection and inflammation.^11,14^ In neonatal populations, there is evidence that decreased HRV correlates with late-onset sepsis,^15^ necrotising enterocolitis^16^ as well as poor long-term outcomes in hypoxic ischaemic encephalopathy.^17^ HRV has also been used to assess autonomic nervous system maturation.^18^

A wide range of HRV metrics is currently in use. The standards of measurement, use and interpretation have been described by The Task Force of The European Society of Cardiology and The North American Society of Pacing and Electrophysiology.^12^ HRV can be characterised in the long-term (up to 24-h recordings), short-term (around 5 min) or ultra-short-term (<5 min).^12,19^ HRV metrics can be categorised into time domain, frequency domain and non-linear measurements. Time domain measurements quantify the variability in the time interval between successive heartbeats using simple equations. The main time domain metrics include the standard deviation of the R-to-R interval (SDNN), the standard deviation of the average R-to-R interval calculated over a given segment (SDANN) or the square root of the mean squared differences of successive N-to-N intervals (RMSSD).^12^ Frequency domain measurements focus on the distribution of absolute or relative power into different frequency bands. Those are the ultra low, very low, low and high-frequency bands.^12^ A further commonly used metric is the total power measurement describing the variance of the N-to-N intervals. Non-linear metrics allow the quantification of unpredictability or self-similarity of the trace.^20,21^ Non-linear metrics commonly used in the literature include the α1 and α2 components obtained by the detrended fluctuation analysis,^22^ SD1 and SD2 parameters obtained by the Poincare analysis, skewness or kurtosis.^21^ Common HRV metrics of all three domains are summarised in Supplementary Material Table 1. An additional metric, heart rate characteristics (HRC), was developed specifically for the detection of neonatal sepsis.^23^ It incorporates multiple other HRV metrics including standard deviation of the inter-heartbeat (RR) intervals, sample asymmetry, sample entropy, and skewness of HR towards frequent large decelerations and few accelerations. These are used to calculate the HeRO score or HRC index, a multivariate logistic regression expression representing the fold increase in risk of sepsis-related clinical deterioration. Low HRV, high sample asymmetry and low entropy result in a higher HeRO score.^23^

Acute and long-term neurological outcomes in preterm infants can be predicted with variable success using different predictive models.^24–29^ At the moment, however, these models rely on MRI findings or patient demographics and circumstances surrounding birth.^27,30,31^ Information on HR and HRV, despite being easily available, is not clinically used for outcome prediction. The present scoping review examines the utility of HRV measurements in the prediction and diagnosis of short-term and long-term neurological outcomes in preterm neonates. The aim of this review is to summarise the available evidence and identify areas in need of further research.

## Methods

A systematic literature search was carried out to identify relevant literature.

### Eligibility

Studies were considered for inclusion only if they included neonates born preterm, defined as <37 weeks of gestational age. Studies that included both term and preterm neonates were only eligible if analysis for both cohorts was separate. Studies focusing on infants with significant congenital defects or chromosomal abnormalities were excluded. Furthermore, studies were only included if a measure of HRV was recorded in the neonatal intensive care unit and during the neonatal period, defined as the first 4 weeks of life. Any measure of HRV allowed inclusion. The last criterion for inclusion was the evaluation of a neurological outcome. Any neurological outcome was considered for inclusion and could be evaluated at any age. Any human observational studies or clinical trials were considered for inclusion. Case reports and case series were excluded.

### Information sources

Medline, Embase, Web of Science and the Cochrane Library were searched on the 3rd of October 2023. No limits were imposed on the date of publication. The search strategy was developed together with the librarian team at the University of Cambridge School of Clinical Medicine and included variations on the term ‘preterm infant’ and ‘heart rate variability’. Neurological outcomes were not explicitly specified in the search strategy. A search strategy used for Embase can be found in the Supplementary Material. Reports were only eligible for inclusion if they were published in the English language to allow for accurate data extraction. Both peer-reviewed articles and conference abstracts were considered for inclusion to minimise the effect of publication bias. All publications citing an included report, and any publications cited by an included report have also been considered for inclusion.

### Data handling

**Fig. 1:**
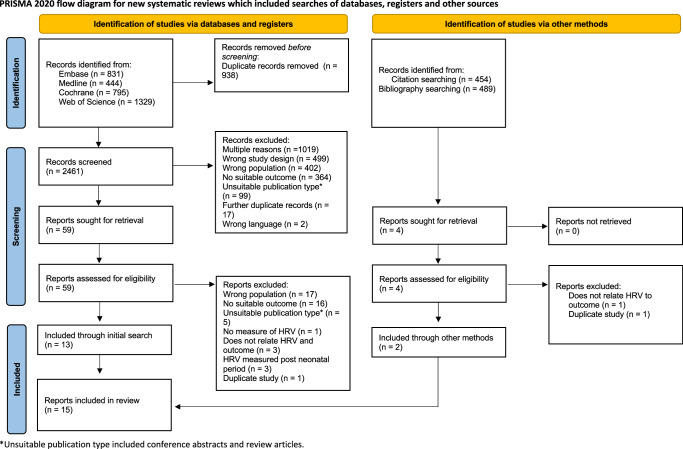
PRISMA 2020 flow diagram for systematic reviews describing the information sources and screening process of the present scoping review. * Unsuitable publication type included conference abstracts and review articles.

Duplicates were removed manually using Zotero,^32^ a reference manager, and Rayyan,^33^ a software designed to simplify screening for systematic reviews. Initial screening was carried out independently by M.S., S.S. and S.H.K. using Rayyan to identify papers suitable for full-text screen. Full-text screen to select papers for inclusion was carried out by S.S. and M.S., with S.H.K. resolving any disagreements. For each study excluded during a full-text screen, a reason is given in the PRISMA 2020 flow chart (Fig. 1).^34^ Data extraction was carried out independently by M.S. and S.S. using a previously piloted data extraction form. All conflicts were settled through discussion or review by S.H.K. or J.S.

Litmaps^35^ software was used to identify further eligible reports amongst the citation network of included reports. Data extraction from these additional papers was carried out independently by SHK and MS.

The following items were extracted from each paper:

Report characteristics:

- Title, author, journal, year of publication, publication type, country of origin, conflict of interest, and sources of funding.

Methods:

- Specific aims of the study, setting, study design.
- Age at HRV collection, infant state during HRV collection, duration of HRV collection, HRV metric used.
- Exclusion and inclusion criteria, number of participants, male to female ratio, gestational age at birth, birth weight, interventions/treatment offered to infants, prevalence of other neonatal conditions.
- Age at outcome assessment, specified neurological outcome, definition of neurological outcome, method of outcome assessment, masking.
- Method of analysis.

Findings:

- Specific findings, missing information, conclusion.

A risk of bias assessment was carried out using the QUADAS-2 tool for the assessment of diagnostic accuracy.^36^

HRV metrics were further classified based on Chiera et al.^21^ as time domain, frequency domain, or non-linear with HRC also being a separate category. Due to the heterogeneity of the data, results were summarised in narrative form.

## Results

After screening 2461 original records, 59 reports were selected to be screened by full text. Thirteen reports were included following the initial search (Fig. 1). Two further reports were identified using Litmaps,^35^ an online tool used to visualise citation links amongst existing literature.

Characteristics, including subject sample size, HRV metrics used, and neurological outcomes, of the included studies are summarised in Table 1. Details of data recording and infant state during recording are shown in Table 2. Eight of the studies were conducted in the United States, and one in each of Finland, Ireland, Australia, Israel, Germany, Japan and the Netherlands. Most studies were prospective cohort or case-control studies. Six studies were retrospective and there were no randomised clinical trials.

**Table 1:** Characteristics of included studies.

Study	Country	Design	*N*	HRV metric	HRV metric category	Neurological outcome	Neurological outcome category	Age at outcome assessment
Addison et al.^46^	USA	Retrospective cohort study	65^b^	cHRC	Heart rate characteristics	1. Cerebral palsy; 2. BSID-II	1. Long term; 2. Long term	12 (*n* = 58) or 18 (*n* = 7) months
Doussard-Roosevelt et al.^48 a^	USA	Prospective cohort study	41	RSA RSA Maturation	Frequency domain	Behavioural parameters determined by scores on Child Behaviour Checklist, Kaufman Assessment Battery for Children, Parenting Stress Index and California Preschool Social Competency Scale	Long term	3 years
Doussard-Roosevelt et al.^47^^ a^	USA	Prospective cohort study	20	RSA RSA Maturation	Frequency domain	Behavioural Parameters determined by scores on Child Behaviour Checklist, Kaufman Assessment Battery for Children, Parenting Stress Index and California Preschool Social Competency Scale	Long term	3 years and school age (mean 8.1 years)
Fairchild et al.^45^	USA	Retrospective cohort study	384^c^	aHRC28	Heart rate characteristics	1. Cranial ultrasound findings; 2. MRI head findings; 3. Bayley scales score	1. Short term; 2. Short term; 3. Long term	1. Twice in first 10 days of life and once after discharge; 2. Term corrected age; 3. 12–15 months of age
Gronlund et al.^37^	Finland	Prospective case-control study	42	RMSSD, RMSM coefficients of variation of RMSSD and RMSM	Time domain	1. IVH grades 1–2 or 3–4; 2. Periventricular leukomalacia	1. Short term; 2. Short term	Days 1–4 of life
Hanna et al.^38^	USA	Retrospective cohort study	19	SDANN index SDNN index	Time domain	1. BSID-II MDI and PDI score; 2. Diagnosis of CP 3. IVH/PVL	1. Long term; 2. Long term; 3. Short term	1. 1 year; 2. 1 year; 3. During admission
Huvanandana et al.^39^	Australia	Prospective case-control study	27	Mean pulse interval short-term and long-term scaling exponents of the pulse interval	Non-linear	IVH	Short term	Two, 12, 24, 36 h and then once daily until the end of the 1st week of life
Hadas et al.^51^	Israel	Prospective cohort study	46	NN, SDNN, RMSSD, LF, HF, TP	Time and frequency domain	1. General movement assessment; 2. Motor optimality score for 3–5-month-old infants	1. Medium term 2. Medium term	1. 35 weeks; 2. 4 months
King et al.^49^	USA	Retrospective cohort study	598	cHRC	Heart Rate Characteristics	Neurodevelopmental impairment (based on blindness, deafness, gross motor function classification system level 2 or higher, or BSID-III cognitive score <85)	Long term	18–22 months corrected age
Lloyd et al.^50^	Ireland	Retrospective cohort study	43	SDNN Skewness Kurtosis	Time domain and non-linear	BSID-III	Long term	2 years of age
Prietsch et al.^40^	Germany	Prospective cohort study	105	Long-term variability and short-term variability	Time domain	IVH	Short term	NA
Shiono et al.^41^	Japan	Prospective case-control study	34^d^	NN SDNN LF HF TP LF/HF CVRR	Time and frequency domain	1. IVH; 2. Neurodevelopmental delay	1. Short term; 2. Long term	1. Within 24 h of birth and then at least every 24 h; 2. 18 months and 3 years corrected age
Sullivan et al.^42^	USA	Retrospective cohort study	566	aHRC-24 h aHRC-7 d	Heart rate characteristics	IVH grades 3–4	Short term	During admission
Tuzcu et al.^43^	USA	Prospective case-control study	24	Detrended fluctuation analysis	Non-linear	IVH	Short term	First week of life
van Ravenswaaij-Arts et al.^44^	Netherlands	Prospective case-control study	50	RR-interval four long-term variability and four short-term variability parameters	Time domain	IVH	Short term	NA

Please refer to Supplementary Table 1 for HRV metric definitions.

*Min* minutes, *H* hours, *D* days, *NA* not specified in report, *HRV* heart rate variability, *BSID-II* Bayley Scales for Infant and Toddler Development Second Edition, *IVH* intraventricular haemorrhage, *MDI* mental developmental index, *PDI* psychomotor developmental index, *CP* cerebral palsy, *BSID-III* Bayley Scales for Infant and Toddler Development Third Edition.

^a^Both studies were describing the same cohort of infants.

^b^One infant did not undergo neurological examination for cerebral palsy at follow-up.

^c^384 was the total number of infants. 50 infants had brain MRI and 70 had BSID data.

^d^Only 23 infants were available for long-term follow-up.

**Table 2:** Description of data recording used by each study.

Study	Length of continuous HRV segment analysed	Infant state during recording	Device used for recording
Addison et al.^46^	>24 h	Long-term continuous recording	ECG
Doussard-Roosevelt et al.^48^^a^	10–15 min	Sleep (not specified)	ECG
Doussard-Roosevelt et al.^47 a^	10–15 min	Sleep (not specified)	ECG
Fairchild et al.^45^	>24 h	Long-term continuous recording	HeRO monitor (using ECG)
Gronlund et al.^37^	2 min	Quiet sleep	ECG
Hanna et al.^38^	20 min	During morning multisensory stimulation (done for a separate study)	NA
Huvanandana et al.^39^	Mean 156 min	NA	Umbilical or peripheral arterial catheter
Hadas et al.^51^	24 h	Long-term continuous recording	ECG
King et al.^49^	NA	NA	HeRO monitor (using ECG)
Lloyd et al.^50^	1 h	NA	NA
Prietsch et al.^40^	>24 h	Long-term continuous recording	Neonatal monitor measuring HR
Shiono et al.^41^	NA	NA	ECG
Sullivan et al.^42^	> 6 h	Long-term continuous recording	HeRO monitor (using ECG)
Tuzcu et al.^43^	~1 h	Supine quiet infants before surfactant administration/tracheal suctioning and then afterwards	ECG
van Ravenswaaij-Arts et al.^44^	3 min	Sleep (not specified)	ECG

*NA* not specified in the report, *ECG* electrocardiogram, *HR* heart rate.

^a^Both studies were conducted in the same cohort of infants.

Four studies measured HRC, seven studies focussed on time domain metrics, four studies on frequency domain metrics, and three on non-linear metrics of HRV. Three studies independently used multiple domains of HRV.

A risk of bias assessment for each study can be found in Supplementary Table 2.

### IVH development may be preceded by a decrease in HRV

Nine studies investigated the relationship between HRV and brain injury, most commonly IVH or WMI.^37–45^ Of these nine studies, all but three studies found an association. This is summarised in Table 3. Six studies revealed a significant decrease in HRV in the context of IVH while two studies found a significant decrease in HRV in infants with WMI. Tuzcu et al.^43^ reported a significant increase in the short-range scaling exponent, a non-linear metric, towards one in infants with IVH. Gronlund et al.^37^ found no association of HRV with IVH or WMI, and van Ravenswaaij-Arts et al.^44^ showed the decrease in HRV in infants with IVH was not statistically significant. Furthermore, three out of the four studies that investigated the temporal relationship between HRV change and brain injury onset found that HRV change preceded visible changes on cranial ultrasound. Prietsch et al.^40^ suggested HRV changes might not precede the development of IVH, but this was based on anecdotal evidence from one patient.

**Table 3:** Findings relating to short-term neurological outcome.

Study	Neurological outcome	Findings	Evidence of IVH prediction
Fairchild et al.^45^	1. Cranial ultrasound findings; 2. MRI Head findings	↑ aHRC28 in infants with ↑ brain injury severity even after adjusting for gestational age, birth weight and late-onset sepsis when compared to controls: 2.65 ± 1.27 vs 1.72 ± 0.95, *p* < 0.001 after multivariate analysis	NA
Gronlund et al.^37^	1. IVH grades 1–2 (2 infants) or 3–4 (10 infants); 2. Periventricular leukomalacia (8 infants)	No association between IVH or periventricular leukomalacia and HRV changes	NA
Hanna et al.^38^	IVH/PVL	↓SDNN index in IVH group (8.4 $\pm$0.6 vs 10.3$\pm$0.5) ↑SDNN index in PVL group (11.7 $\pm$0.6 vs 10.3 $\pm$0.5) *p* = 0.004	NA
Huvanandana et al.^39^	IVH	HRV parameters did not correlate with IVH by themselves but combination of long-term fractal exponent of the pulse interval with mean diastolic blood pressure was able to predict IVH with a sensitivity of over 90% and better than all other models, AUC (95% CI) = 0.921 (0.82, 1.02), *p* = 0.035.	This model was able to predict IVH
Prietsch et al.^40^	IVH	↓LTV correlated with IVH development 5/7 of the patients in the IVH group subsequently died	Anecdotal evidence from one patient shows normal LTV which then decreased just prior to IVH diagnosis, implying HRV changes do not precede IVH
Shiono et al.^41^	IVH	↓NN interval on days 1, 2 and 3, ↓ SDNN and CVRR on day 4, ↓ TP and LF on days 3, 4 and 5 and ↓ HF and LF/HF on days 4 and 5 in infants with grade IV IVH (*p* < 0.05 for all observations stated) Grades I–II IVH infants did not significantly differ from controls	NA
Sullivan et al.^42^	IVH grades 3–4	Severe IVH was associated with ↑aHRC-24 h and ↑ aHRC-7 d even after accounting for gestational age (*p* ≤ 0.01)	Report implies HRC changes predict IVH occurrence
Tuzcu et al.^43^	IVH	↑ short-range scaling exponent calculated for 8–15 beats in infants with IVH. Cut-off value of 0.52 (corresponding to predicted probability of 0.42) resulted in 70% sensitivity and 79% specificity in detecting IVH No other measured parameters were significantly different	HRV alteration was apparent on day 1 when cranial ultrasound was normal
van Ravenswaaij-Arts et al.^44^	IVH	↓ short-term variability in infants with IVH (ns)^a^	NA

Please refer to Supplementary Table 1 for heart rate variability metric definitions.

*NA* not specified in report, *ns* not significant, *IVH* intraventricular haemorrhage, *PVL* periventricular leukomalacia, *HRV* heart rate variability.

^a^Short-term variability was defined using four different methods in this paper and the method(s) used to reach this specific conclusion was not specified.

### Decreased HRV correlates with impaired long-term neurodevelopmental outcomes

Eight studies related the long-term neurodevelopmental outcome of infants with recorded HRV.^38,45–50^ All eight papers found an association between abnormal HRV measurement and the development of neurological impairment later in life. This is summarised in Table 4. Associations were found between various domains of neurological function and various metrics of HRV. In general, HRV was lower or failed to increase in infants who went on to develop neurological impairment.

**Table 4:** Findings relating to long-term neurological outcomes.

Study	Neurological outcome	Findings	Parameters
Addison et al.^46^	1. Cerebral palsy 2. BSID-II	1. ↑ cHRC in infants with cerebral palsy (*p* < 0.01) 2. ↑ cHRC in infants with MDI < 70 or PDI < 70 (*p* < 0.01)	Odds ratio of cerebral palsy per one standard deviation increase in cHRC: 2.6; odds ratio of delayed early cognitive development: 2.3
Doussard-Roosevelt et al.^48^^ a^	Behavioural parameters determined by scores on Child Behaviour Checklist, Kaufman Assessment Battery for Children, Parenting Stress Index and California Preschool Social Competency Scale	RSA maturation was correlated with mental processing (0.62, *p* < 0.01), knowledge base (0.55, *p* < 0.05) and gross motor skills (0.47, *p* < 0.05) in very low birth weight infants but not extremely low birth weight infants. Mental processing (0.38, *p* < 0.05) and gross motor skills (0.37, *p* < 0.05) were still correlated when the two groups were combined. RSA maturation and HR maturation together were correlated with behavioural regulation.	
Doussard-Roosevelt et al.^47^^a^	Behavioural parameters determined by scores on Child Behaviour Checklist, Kaufman Assessment Battery for Children, Parenting Stress Index and California Preschool Social Competency Scale	Greater maturation of RSA in NICU was associated with better social competence at school age (0.54, *p* < 0.05). Together with medical risk and socioeconomic status, they accounted for 58% of variability in social competence. No significant correlation with behavioural problems, Parenting Stress Index, Kaufman Assessment Battery for Children Achievement Scale or Mental Processing Composite Scale.	
Fairchild et al.^45^	BSID-III	↑ aHRC28 in infants with any BSID-III score <70	A one-point increase in aHRC28 corresponded to an odds ratio of death beyond 28 days or BSID-III Score <70 of 2.45
Hanna et al.^38^	1. BSID-II MDI and PDI score; 2. Diagnosis of cerebral palsy	↑SDNN, ↑ SDNN index and ↑ RMSD correlated with improved MDI score in healthy infants but not brain injured ones (*p* < 0.04)	
King et al.^49^	Neurodevelopmental impairment (based on blindness, deafness, gross motor function classification system level 2 or higher, or BSID-III cognitive score <85)	↑ cmHRC in infants with moderate-to-severe NDI when compared to those with no or mild NDI ↑ Daily HRC scores in infants who died or developed than in patients without NDI from the 1st postnatal day to day 120 when data collection ended	Severe impairment had cmHRC of 1.8 ± 1.1, good outcome 1.3 ± 0.8, statistically significant
Lloyd et al.^50^	BSID-III score	↓HR skewness correlates with poor neurodevelopmental outcomes Multimodal information had a higher area under the receiver operator characteristic so it may be more useful to integrate HRV with other information	Univariate regression model with sensitivity 70%, specificity 69%, PPV 79%, NPV 58%
Shiono et al.^41^	Neurodevelopmental delay	↓HRV (CVRR, SDNN, TP, LF, HF, LF/HF) was related to an increased risk of adverse neurodevelopmental outcomes at 18 months	Does not clarify whether this is statistically significant

Please refer to Supplementary Table 1 for heart rate variability metric definitions.

*NA* not specified in report, *BSID-II* Bayley Scales for Infant and Toddler Development Second Edition, *MDI* mental developmental index, *PDI* psychomotor developmental index, *RSA* respiratory sinus arrhythmia, *HR* heart rate, *BSID-III* Bayley Scales for Infant and Toddler Development Third Edition, *NDI* neurodevelopmental impairment, *HRV* heart rate variability, *PPV* positive predictive value, *NPV* negative predictive value.

^a^Both studies were describing the same cohort of infants.

### Other outcomes investigated

Hadas et al. investigated the relationship between HRV and motor outcomes at 35 weeks and 4 months corrected age.^51^ Using time domain and frequency domain metrics, they found that decreased HRV correlated with suboptimal motor outcomes at both ages.

## Discussion

The present evidence suggests an association between HRV and short-term and long-term neurological outcomes in preterm infants.

### Utility of HRV monitoring in brain injury detection and prediction

Seven out of nine studies suggest an association between decreasing HRV and brain injury. The brain injury most commonly studied was IVH with all nine studies including IVH as an outcome. Three studies also considered the diagnosis of WMI.

In terms of metrics used, studies were able to detect an association with HRC, time domain, non-linear and frequency domain metrics. Interestingly, studies that failed to find an association all relied on time domain metrics. Amongst the four studies using time domain, only half detected an association. This may suggest that time domain is less well suited for the detection of brain injury. A similar phenomenon has been proposed by the authors of the HeRO score, who found the frequency domain to be less useful than other metrics in the prediction of neonatal sepsis.^23^

Furthermore, there was great variation in the time intervals used for HRV analysis. Studies that found no association between HRV and brain injury, including Gronlund et al.,^37^ and van Ravenswaaij-Arts et al.,^44^ all relied on shorter HRV segments than every other study. The time intervals used in this case were 2 and 3 min, respectively. Time intervals used by studies that found a significant difference in HRV ranged from over 15 min to over 24 h (Table 2). The length of the recording was not specified by Shiono et al.^41^ This implies that short-term and long-term HRV may be better predictors of brain injury than ultra-short-term HRV.

Moreover, HRV changes were more closely associated to higher grade IVH. Shiono et al.^41^ and Fairchild et al.^45^ found HRV changes in infants with high-grade IVH but not infants with grades 1–2. Gronlund et al.^37^ and van Ravenswaaij-Arts et al.^44^ both investigated infants with IVH of all grades and failed to find a significant difference in HRV. Other studies that specified IVH grade seem to have focused on high-grade IVH (Table 3). This suggests that HRV monitoring may not be sensitive enough to detect low-grade IVH.

Four studies specifically investigated the temporal relationship between the onset of brain injury. All four of those studies were able to detect a change in HRV prior to the detection of brain injury on cranial ultrasound. This suggests that HRV monitoring may be useful in very early prediction and might allow for preventive measures that can reduce the risk of injury.^52^

Fewer studies overall investigated the relationship between HRV and WMI. Fairchild et al.^45^ reported an increase in HRC in moderate-to severe WMI, Gronlund et al.^37^ found no association and Hanna et al.^38^ reported an increase in HRV (as opposed to a decrease seen in infants with IVH). Overall, there does not seem to be a clearcut relationship between HRV and WMI in preterm infants.

All in all, the data suggests that HRV has potential role in the early prediction of IVH, especially higher grades, in preterm infants.

### Utility of HRV monitoring in the prediction of long-term neurodevelopmental outcomes

Eight studies, utilising time domain, frequency domain, non-linear and HRC metrics of long-term, short-term and ultra-short-term variability, all found an association between decreased HRV and adverse neurodevelopmental outcomes. Outcomes were most commonly assessed using The Bayley Scales of Infant and Toddler Development—third edition (BSID-III)^53^ or second edition (BSID-II) scores. Some studies used the presence and absence of CP, visual or hearing impairment, or questionnaires. Amongst the questionnaires used were the Child Behavioural Checklist,^54^ Kaufman Assessment Battery for Children,^55^ Parenting Stress Index^56^ and California Preschool Social Competency Scale.^57^ In these studies, a decrease in HRV has been correlated with poor psychomotor development, mental development, social development and increased rates of intellectual disability. There seems to be no domain that is better correlated than other. This suggests that HRV during the neonatal period may be an indicator of impending global developmental delay.

### Limitations of the literature

One of the main limitations of the present review derives from the large diversity in the HRV metrics and the large diversity in the reporting of neurodevelopmental outcomes. While many studies use predefined scales, several studies used ambiguous neurological outcomes as defined by the neurologist or neonatologist. The length of recording and specific setting of HRV recording also differs greatly between the studies with the shortest recordings being just 2 min while the longest are over 168 h. Furthermore, the state of the infant at recording, which determines a large proportion of HRV at any given moment, is often omitted in the methods sections or only loosely defined as ‘sleep’ or ‘no movement artefacts’.^58^ The effect of infant state is minimised by using long-term variability measurements spanning across multiple sleep–wake cycles. This was however not the norm amongst the studies included. Moreover, the limited number and categories of metrics used by each study prevented valid comparisons between metrics.

### Limitations of the present review

The present review was carried out and reported in line with the PRISMA Extension for Scoping Reviews Guidelines.^34^ The entire body of work was completed within 9 months of the literature search to capture all the relevant literature. Moreover, the search strategy was designed to capture a greater body of literature by not limiting it to records mentioning neurological outcomes. This allowed us to assess the outcomes of any screened study individually and likely resulted in more eligible studies being identified.

Due to a large variability in reporting of neurological outcomes and HRV metrics, we do not believe it is currently possible or useful to conduct a systematic review or meta-analysis on the topic. This also means we were unable to carry out any quantitative analysis of the results.

Nonetheless, we believe our search strategy captured most of the available papers as we were only able to identify two further studies through secondary search methods.

### Further work

Current literature supports a strong association between changes in HRV and IVH development. Importantly, HRV can be extracted from ECG traces which are obtained routinely for preterm neonates. No further intervention to the infant would therefore be required to implement HRV screening on the NICU. Because of this, further research has the potential to have a significant impact on patient care. In order for HRV to be used clinically, the relationship between IVH and HRV needs to be further evaluated to determine which metrics have the most prognostic value. It would also be useful to determine the length of trace required for analysis. The next important step would be to determine whether early detection or even prediction of IVH and intervention in preterm infants with brain injury using HRV monitoring has the potential to improve outcomes. Further studies focusing on the detection of WMI in preterm infants using HRV would be beneficial, as the relationship remains unclear.

In summary, while the present data suggest reduced HRV correlates with brain injury and long-term neurodevelopmental impairment, more research is required to confirm and further quantify this relationship.

## Supplementary information

Embase search strategy

PRISMA Checklist

Supplementary table 1

Supplementary table 2

## References

[1] Platt, M. J. Outcomes in preterm infants. *Public Health* **128**, 399–403 (2014). 24794180 10.1016/j.puhe.2014.03.010

[2] *Preterm Birth: Causes, Consequences, and Prevention* (Washington, DC, 2007). 10.17226/11622. 20669423

[3] Inder, T. E., Volpe, J. J. & Anderson, P. J. Defining the neurologic consequences of preterm birth. *N. Engl. J. Med.* **389**, 441–453 (2023). 37530825 10.1056/NEJMra2303347

[4] Ballabh, P. & de Vries, L. S. White matter injury in infants with intraventricular haemorrhage: mechanisms and therapies. *Nat. Rev. Neurol.* **17**, 199–214 (2021). 33504979 10.1038/s41582-020-00447-8 PMC8880688

[5] Delobel-Ayoub, M. et al. Behavioral outcome at 3 years of age in very preterm infants: the EPIPAGE study. *Pediatrics* **117**, 1996–2005 (2006). 16740841 10.1542/peds.2005-2310

[6] Aylward, G. P. Neurodevelopmental outcomes of infants born prematurely. *J. Dev. Behav. Pediatr.* **26**, 427–440 (2005). 16344661 10.1097/00004703-200512000-00008

[7] Anderson, P. J., Doyle, L. W. & Victorian Infant Collaborative Study Group. Executive functioning in school-aged children who were born very preterm or with extremely low birth weight in the 1990s. *Pediatrics* **114**, 50–57 (2004). 15231907 10.1542/peds.114.1.50

[8] Saigal, S. & Doyle, L. W. An overview of mortality and sequelae of preterm birth from infancy to adulthood. *Lancet* **371**, 261–269 (2008). 18207020 10.1016/S0140-6736(08)60136-1

[9] Cizmeci, M. N. et al. Randomized controlled early versus late ventricular intervention study in posthemorrhagic ventricular dilatation: outcome at 2 years. *J. Pediatr.* **226**, 28–35.e3 (2020). 32800815 10.1016/j.jpeds.2020.08.014

[10] Spittle, A., Orton, J., Anderson, P. J., Boyd, R. & Doyle, L. W. Early developmental intervention programmes provided post hospital discharge to prevent motor and cognitive impairment in preterm infants. *Cochrane Database Syst. Rev.*10.1002/14651858.CD005495.pub4 (2015). 10.1002/14651858.CD005495.pub4 PMC8612699 26597166

[11] Tiwari, R., Kumar, R., Malik, S., Raj, T. & Kumar, P. Analysis of heart rate variability and implication of different factors on heart rate variability. *Curr. Cardiol. Rev.* **17**, e160721189770 (2021). 33390146 10.2174/1573403X16999201231203854 PMC8950456

[12] Task Force of the European Society of Cardiology and the North American Society of Pacing and Electrophysiology. Heart rate variability: standards of measurement, physiological interpretation and clinical use. *Circulation* **93**, 354–381 (1996). 8737210

[13] Jarczok, M. N. et al. Heart rate variability in the prediction of mortality: a systematic review and meta-analysis of healthy and patient populations. *Neurosci. Biobehav. Rev.* **143**, 104907 (2022). 36243195 10.1016/j.neubiorev.2022.104907

[14] Bravi, A. et al. Do physiological and pathological stresses produce different changes in heart rate variability? *Front. Physiol.* **4**, 197 (2013). 23908633 10.3389/fphys.2013.00197 PMC3726831

[15] Fairchild, K. & O’Shea, T. Heart rate characteristics: physiomarkers for detection of late-onset neonatal sepsis. *Clin. Perinatol.* **37**, 581 (2010). 20813272 10.1016/j.clp.2010.06.002 PMC2933427

[16] Stone, M. et al. Abnormal heart rate characteristics before clinical diagnosis of necrotizing enterocolitis. *J. Perinatol.* **33**, 847–850 (2013). 23722974 10.1038/jp.2013.63 PMC4026091

[17] Bersani, I. et al. Heart rate variability as possible marker of brain damage in neonates with hypoxic ischemic encephalopathy: a systematic review. *Eur. J. Pediatr.* **180**, 1335–1345 (2021). 33245400 10.1007/s00431-020-03882-3 PMC7691422

[18] Patural, H., Pichot, V., Roche, F. & Giraud, A. Why, when and how to assess autonomic nervous system maturation in neonatal care units: A practical overview. *Neurophysiol. Clin.* **53**, 102855 (2023). 36965238 10.1016/j.neucli.2023.102855

[19] Orini, M. et al. Long-term association of ultra-short heart rate variability with cardiovascular events. *Sci. Rep.* **13**, 18966 (2023). 37923787 10.1038/s41598-023-45988-2 PMC10624663

[20] Shaffer, F. & Ginsberg, J. P. An overview of heart rate variability metrics and norms. *Front. Public Health* **5**, 258 (2017). 10.3389/fpubh.2017.00258 PMC5624990 29034226

[21] Chiera, M. et al. Heart rate variability in the perinatal period: a critical and conceptual review. *Front. Neurosci.* **14**, 561186 (2020). 10.3389/fnins.2020.561186 PMC7544983 33071738

[22] Peng, C.-K., Havlin, S., Stanley, H. E. & Goldberger, A. L. Quantification of scaling exponents and crossover phenomena in nonstationary heartbeat time series. *Chaos Interdiscip. J. Nonlinear Sci.* **5**, 82–87 (1995). 10.1063/1.166141 11538314

[23] Fairchild, K. D. Predictive monitoring for early detection of sepsis in neonatal ICU patients. *Curr. Opin. Pediatr.* **25**, 172 (2013). 23407184 10.1097/MOP.0b013e32835e8fe6 PMC10989716

[24] Kumar, P. & Polavarapu, M. A simple scoring system for prediction of IVH in very-low-birth-weight infants. *Pediatr. Res.* **94**, 2033–2039 (2023). 37479747 10.1038/s41390-023-02744-6

[25] van Boven, M. R. et al. Machine learning prediction models for neurodevelopmental outcome after preterm birth: a scoping review and new machine learning evaluation framework. *Pediatrics* **150**, e2021056052 (2022). 35670123 10.1542/peds.2021-056052

[26] Cayam-Rand, D. et al. Predicting developmental outcomes in preterm infants. *Neurology* **93**, e1231–e1240 (2019). 31467250 10.1212/WNL.0000000000008172 PMC7011867

[27] Routier, L. et al. Predicting the neurodevelopmental outcome in extremely preterm newborns using a multimodal prognostic model including brain function information. *JAMA Netw. Open* **6**, e231590 (2023). 36884252 10.1001/jamanetworkopen.2023.1590 PMC9996404

[28] Juul, S. E. et al. Predicting 2-year neurodevelopmental outcomes in extremely preterm infants using graphical network and machine learning approaches. *eClinicalMedicine* **56**, 101782 (2023). 10.1016/j.eclinm.2022.101782 PMC9813758 36618896

[29] Moeskops, P. et al. Prediction of cognitive and motor outcome of preterm infants based on automatic quantitative descriptors from neonatal MR brain images. *Sci. Rep.* **7**, 2163 (2017). 28526882 10.1038/s41598-017-02307-w PMC5438406

[30] Martini, S. et al. Neurodevelopmental correlates of brain magnetic resonance imaging abnormalities in extremely low-birth-weight infants. *J. Pediatr.* **262**, 113646 (2023). 10.1016/j.jpeds.2023.113646 37516269

[31] Jang, Y. H. et al. Predicting 2-year neurodevelopmental outcomes in preterm infants using multimodal structural brain magnetic resonance imaging with local connectivity. *Sci. Rep.* **14**, 9331 (2024). 38653988 10.1038/s41598-024-58682-8 PMC11039622

[32] Zotero (Corporation for Digital Scholarship, 2024).

[33] Ouzzani, M., Hammady, H., Fedorowicz, Z. & Elmagarmid, A. Rayyan—a web and mobile app for systematic reviews. *Syst. Rev.* **5**, 210 (2016). 27919275 10.1186/s13643-016-0384-4 PMC5139140

[34] Tricco, A. C. et al. PRISMA extension for scoping reviews (PRISMA-ScR): checklist and explanation. *Ann. Intern. Med.* **169**, 467–473 (2018). 30178033 10.7326/M18-0850

[35] Litmaps. https://www.litmaps.com/ (2024).

[36] Whiting, P. F. et al. QUADAS-2: a revised tool for the quality assessment of diagnostic accuracy studies. *Ann. Intern. Med.* **155**, 529–536 (2011). 22007046 10.7326/0003-4819-155-8-201110180-00009

[37] Gronlund, J. U., Korvenranta, H., Kero, P., Jalonen, J. & Valimaki, I. A. Elevated arterial blood pressure is associated with peri-intraventricular haemorrhage. *Eur. J. Pediatr.* **153**, 836–841 (1994). 7843200 10.1007/BF01972894

[38] Hanna, B. D. et al. Heart rate variability in preterm brain-injured and very-low-birth-weight infants. *Biol. Neonate* **77**, 147–155 (2000). 10729717 10.1159/000014209

[39] Huvanandana, J. et al. Prediction of intraventricular haemorrhage in preterm infants using time series analysis of blood pressure and respiratory signals. *Sci. Rep.* **7**, 46538 (2017). 10.1038/srep46538 PMC5402275 28436467

[40] Prietsch, V., Knoepke, U. & Obladen, M. Continuous monitoring of heart rate variability in preterm infants. *Early Hum. Dev.* **37**, 117–131 (1994). 8088228 10.1016/0378-3782(94)90153-8

[41] Shiono, A. et al. Autonomic nervous system in preterm very low birth weight neonates with intraventricular hemorrhage. *Am. J. Perinatol*. 10.1055/a-1926-0335 (2022). 10.1055/a-1926-0335 35977712

[42] Sullivan, B. A. et al. Early heart rate characteristics predict death and morbidities in preterm infants. *J. Pediatr.* **174**, 57–62 (2016). 27113378 10.1016/j.jpeds.2016.03.042 PMC5672906

[43] Tuzcu, V., Nas, S., Ulusar, U., Ugur, A. & Kaiser, J. R. Altered heart rhythm dynamics in very low birth weight infants with impending intraventricular hemorrhage. *Pediatrics* **123**, 810–815 (2009). 19255007 10.1542/peds.2008-0253 PMC2871543

[44] van Ravenswaaij-Arts, C. M. A. et al. The influence of respiratory distress syndrome on heart rate variability in very preterm infants. *Early Hum. Dev.* **27**, 207–221 (1991). 1802672 10.1016/0378-3782(91)90195-9

[45] Fairchild, K. D. et al. Abnormal heart rate characteristics are associated with abnormal neuroimaging and outcomes in extremely low birth weight infants. *J. Perinatol.* **34**, 375–379 (2014). 24556979 10.1038/jp.2014.18 PMC11019753

[46] Addison, K., Griffin, M. P., Moorman, J. R., Lake, D. E. & O’Shea, T. M. Heart rate characteristics and neurodevelopmental outcome in very low birth weight infants. *J. Perinatol.* **29**, 750–756 (2009). 19554011 10.1038/jp.2009.81 PMC2834345

[47] Doussard-Roosevelt, J., McClenny, B. & Porges, S. Neonatal cardiac vagal tone and school-age developmental outcome in very low birth weight infants. *Dev. Psychobiol.* **38**, 56–66 (2001). 11150061

[48] Doussard-Roosevelt, J. A., Porges, S. W., Scanlon, J. W., Alemi, B. & Scanlon, K. B. Vagal regulation of heart rate in the prediction of developmental outcome for very low birth weight preterm infants. *Child Dev.* **68**, 173–186 (1997). 9179997

[49] King, W. E. et al. Multivariable predictive models of death or neurodevelopmental impairment among extremely low birth weight infants using heart rate characteristics. *J. Pediatr.* **242**, 137–144.e4 (2022). 34798080 10.1016/j.jpeds.2021.11.026

[50] Lloyd, R. et al. Predicting 2-y outcome in preterm infants using early multimodal physiological monitoring. *Pediatr. Res.* **80**, 382–388 (2016). 27089498 10.1038/pr.2016.92

[51] Hadas, I. M., Joseph, M., Luba, Z. & Michal, K. L. Assessing parasympathetic measures of heart rate variability shortly after birth to predict motor repertoire at four months in low risk preterm infants born between 28 and 32 weeks of gestation. *Early Hum. Dev.* **161**, 105438 (2021). 34392066 10.1016/j.earlhumdev.2021.105438

[52] Yao, S. L., Smit, E. & Odd, D. The effectiveness of interventions to prevent intraventricular haemorrhage in premature infants: a systematic review and network meta-analysis. *J. Neonatal-Perinat. Med.* **16**, 5–20 (2023). 10.3233/NPM-221048 36591663

[53] Del Rosario, C., Slevin, M., Molloy, E. J., Quigley, J. & Nixon, E. How to use the Bayley scales of infant and toddler development. *Arch. Dis. Child. Educ. Pract. Ed.* **106**, 108–112 (2021). 32859738 10.1136/archdischild-2020-319063

[54] Achenbach, T. M. & Ruffle, T. M. The Child Behavior Checklist and related forms for assessing behavioral/emotional problems and competencies. *Pediatr. Rev.* **21**, 265–271 (2000). 10922023 10.1542/pir.21-8-265

[55] Kaufman, A. S. & Kaufman, N. L. Kaufman assessment battery for children. 10.1037/t27677-000 (1983).

[56] Abidin, R. R. *Parenting Stress Index: Manual, Administration Booklet, [and] Research Update* (Pediatric Psychology Press, 1983).

[57] Proger, B. B. Test review no. 17: California preschool social competency scale. *J. Spec. Educ.* **8**, 391–395 (1974).

[58] de Groot, E. R. et al. The value of cardiorespiratory parameters for sleep state classification in preterm infants: a systematic review. *Sleep. Med. Rev.* **58**, 101462 (2021). 33826975 10.1016/j.smrv.2021.101462

