# Supplementary material for: Using heart rate variability to predict neurological outcomes in preterm infants: a scoping review
Source: Pediatr Res. 2024 Oct 5;97(6):1823–32. doi: 10.1038/s41390-024-03606-5 (PMC12122357; doi:10.1038/s41390-024-03606-5)
Supplement: Supplementary file 1 — Embase search strategy [file 41390_2024_3606_MOESM1_ESM.docx]

The search strategy used for Embase is shown below:

Embase <1974 to 2023 October 03>

1 (((preterm* or prematur* or early or <37 week or low gestational age) adj3 (infant* or neonat* or baby or babies or newborn*)) or prematurity).mp. [mp=title, abstract, heading word, drug trade name, original title, device manufacturer, drug manufacturer, device trade name, keyword heading word, floating subheading word, candidate term word]

2 ("heart rate variability" or "heart rate characteristics" or HRV).mp.

3 exp Infant, Premature/

4 ((heart or electrocardiogram or ECG) and ("NN interval" or "RR interval" or normal-to-normal or R-wave-to-R-wave or "frequency domain" or power or "total power" or "low frequency" or "LF/HF" or "low-frequency" or unpredictability or self-similarity or chaos)).mp. [mp=title, abstract, heading word, drug trade name, original title, device manufacturer, drug manufacturer, device trade name, keyword heading word, floating subheading word, candidate term word]

5 1 or 3

6 2 or 4

7 5 and 6
