# Supplementary material for: Using heart rate variability to predict neurological outcomes in preterm infants: a scoping review
Source: Pediatr Res. 2024 Oct 5;97(6):1823–32. doi: 10.1038/s41390-024-03606-5 (PMC12122357; doi:10.1038/s41390-024-03606-5)
Supplement: Supplementary file 4 — Supplementary table 2 [file 41390_2024_3606_MOESM4_ESM.docx]

Supplementary Table 2 – Quality assessment using the QUADAS-2 scale

|  | **Patient Selection** | | **Index test** | | **Reference standard** | | **Flow and timing** |
| --- | --- | --- | --- | --- | --- | --- | --- |
| **Study** | **Bias** | **Applicability** | **Bias** | **Applicability** | **Bias** | **Applicability** | **Bias** |
| Addison et al. 2009^48^ | low | low | low | low | low | low | low |
| Doussard-Roosevelt et al. 1997^50^ | low | low | high | low | low | low | high |
| Doussard-Roosevelt et al. 2001^49^ | low | low | high | low | low | low | high |
| Fairchild et al. 2014^46^ | low | low | low | low | low | low | low |
| Gronlund et al. 1994^38^ | low | low | low | low | low | low | low |
| Hadas et al. 2021^53^ | low | low | low | low | low | low | low |
| Hanna et al. 2000^39^ | unclear | low | low | unclear | low | low | low |
| Huvanandana et al. 2017^40^ | low | low | low | low | low | low | low |
| King et al. 2022^51^ | low | low | unclear | low | low | low | high |
| Lloyd et al. 2016^52^ | low | low | low | low | low | low | low |
| Prietsch et al. 1994^41^ | low | high | low | low | low | low | low |
| Shiono et al. 2022^42^ | unclear | low | unclear | low | low | low | low |
| Sullivan et al. 2016^43^ | low | low | low | low | low | low | low |
| Tuzcu et al. 2009^44^ | low | low | low | low | low | low | low |
| van Ravenswaaij-Arts et al. 1991^45^ | low | low | high | low | low | low | high |
